# Olfactory Orientation and Navigation in Humans

**DOI:** 10.1371/journal.pone.0129387

**Published:** 2015-06-17

**Authors:** Lucia F. Jacobs, Jennifer Arter, Amy Cook, Frank J. Sulloway

**Affiliations:** Department of Psychology, University of California, Berkeley, California, United States of America; Center for Genomic Regulation, SPAIN

## Abstract

Although predicted by theory, there is no direct evidence that an animal can define an arbitrary location in space as a coordinate location on an odor grid. Here we show that humans can do so. Using a spatial match-to-sample procedure, humans were led to a random location within a room diffused with two odors. After brief sampling and spatial disorientation, they had to return to this location. Over three conditions, participants had access to different sensory stimuli: olfactory only, visual only, and a final control condition with no olfactory, visual, or auditory stimuli. Humans located the target with higher accuracy in the olfaction-only condition than in the control condition and showed higher accuracy than chance. Thus a mechanism long proposed for the homing pigeon, the ability to define a location on a map constructed from chemical stimuli, may also be a navigational mechanism used by humans.

## Introduction

There are two very different ways to use odors to orient in space. The first is odor tracking, where an animal tracks an odor to its source. This behavior has been demonstrated in walking, flying and swimming invertebrate species [1–3] as well as walking, flying and swimming vertebrates [4–6]. Even blindfolded humans can crawl and track an odor across grass, and their accuracy in tracking decreases if they are deprived of bilateral nostril input [7]. Orienting more accurately with stereo-olfaction is evidence that an animal is using odors to compute the direction to the source [8–10]. Blindfolded humans can also localize an odor’s source by sampling from a central, sitting location [11].

The second way to use odors is to detect the statistical regularities of airborne odors distributed above a landscape and to use this information to define a terrestrial location. This behavior is much less well understood in humans or any other species, although there are many anecdotes of such mapping from sailors, hunters, and early aviators [12]. The idea of atmospheric odors providing stable spatial landmarks was first proposed for the homing pigeon by Floriano Papi in the 1970’s, conceptualized as a mosaic of odor patches [13]. Later, Wallraff proposed that pigeons could also orient to odor gradients organized in a coordinate system. He has since shown, using data from the sampling of atmospheric odors and computational models, that such stable odor gradients could exist and provide sufficient information to explain the navigational accuracy seen in pigeons [14–17].

Strong evidence for olfactory navigation in homing pigeons continues to accumulate, and has accelerated with the invention of global positioning systems [18]. The neural basis for olfactory navigation in pigeons has also been identified; the olfactory bulb and hippocampus are key brain substrates for navigation, both in pigeons and mammals [19,20]. Despite the obvious structural and functional connections between the hippocampus and the olfactory system in mammals [21], studies of hippocampal function, even in rodents, use odors only as identifiers of objects, i.e., as an associative cue [22]. What is rarely manipulated is the use of an extended gradient of odors as an orientation cue, though there are exceptions [23–25]. Odors in rodent studies are thus mostly used in two ways: as a unique feature of a location or object and as a rodent’s orientation to naturally deposited odors—either volitional scent marks or passive odor trails created by the animal’s feet on the apparatus surface. It is unclear whether any study of rodents has required them to navigate to a diffuse odor landscape. However, recently Zhang and Manahan-Vaughn have shown that hippocampal place cells in the rat can be controlled by the position of odors emanating from the four corners of a rectangular space. These hippocampal place fields then rotated when the odors were rotated, and remapped when the odor array was jumbled [26]. This suggests that the rats may have been orienting to a grid formed from the experimental odors.

Because the bird and the mammalian hippocampus are homologous and because both mediate spatial navigation [19], it seems likely that the hippocampus in both groups could be encoding odors as a multi-coordinate bearing map, as originally proposed in the parallel map theory of hippocampal function. In this hypothesis, the cognitive map is unpacked into two maps, one ancestral to all vertebrates (the bearing map) and one map more recently evolved (the sketch map), and which is found only in mammals and birds. The bearing map is proposed as a low resolution grid map, built from extended stimuli such as gradients; the sketch maps are high resolution topological maps that represent arrays of local landmarks. The two maps can be used independently for orientation but must be combined into a single map (the integrated map) for complex navigational problems [27].

We have recently proposed that certain characteristics of the vertebrate main olfactory system, such as the allometry of the olfactory bulb relative to brain size and the psychophysics of odor mixture perception, suggest that many vertebrates, not just birds, map locations in space using odor gradients [21]. If such navigation is a key function of olfaction, then the ability to encode an arbitrary location as a position on a bearing map constructed from odor gradients should therefore be widespread among vertebrates.

One reason that this hypothesis has not been tested, and no doubt an underlying reason for the continuing controversy on this subject [17,28], is the difficulty of measuring and manipulating the distribution of odors, particularly those in the atmosphere. For this reason there is currently no direct evidence that a bird, or indeed any species, can map an arbitrary location in the atmosphere, using the coordinates defined by an olfactory bearing or grid map. Because we predicted that this ability should be widespread in animals, we designed the current study to test this basic assumption of olfactory navigation not in a flying bird but in a terrestrial mammal. Employing a spatial match-to-sample design, we tested this prediction in humans.

## Materials and Methods

The goal of this study was to determine if a person could map an arbitrary location in space using odor alone. Accuracy using only odors was compared to accuracy when study participants were unable to use olfaction, vision, or audition. We also included controls for a variety of potential confounding effects, such as individual differences in navigational ability, key features of the task environment and demographic variables.

### Participants

Participants were 45 undergraduate students (19 men and 26 women) recruited from college classes, who participated in exchange for course credit. Eight additional participants were excluded from data analysis. Four participants were excluded due to technical error during data collection (i.e., either odors were not present in the room during the experimental condition, or research assistants spoke aloud while the participant was standing at the target location in one of the conditions, potentially giving the participant an auditory cue regarding location). One participant was excluded because he or she did not complete the self-report measures included in the study and another was excluded because he or she did not speak English with enough proficiency to understand the instructions. Finally, two participants were excluded because they reported having no current ability to detect odors, owing to illness or allergies. Participants’ average age was 21.8 years (*SD* = 3.3) and reported ethnicities were 21 Asian, 7 Hispanic/Latino, 6 White, and 11 Other or a mixed ethnicity. This research was approved by the Committee for the Protection of Human Subjects, the Institutional Review Board for the University of California, Berkeley.

### Measures

Before participating in the navigation tasks, participants completed a number of self-report measures. Participants reported on their sense of direction via the Santa Barbara Sense of Direction Scale [29]. This scale has fifteen items, seven of which are phrased positively (e.g., “I am very good at giving directions”) and eight of which are phrased negatively (e.g., “I don’t enjoy giving directions”). Participants reported on each item using a 7-point Likert scale (1 = strongly disagree; 7 = strongly agree). Negatively worded items were reverse-coded such that a high score indicated a stronger self-reported sense of direction. Participants also reported on basic demographics, including age, gender, socioeconomic status, political orientation, and sexual orientation, as well as reporting their sibling order status. Female participants reported on their birth control status and menstrual cycle phase.

### Task environment

**Fig 1:**
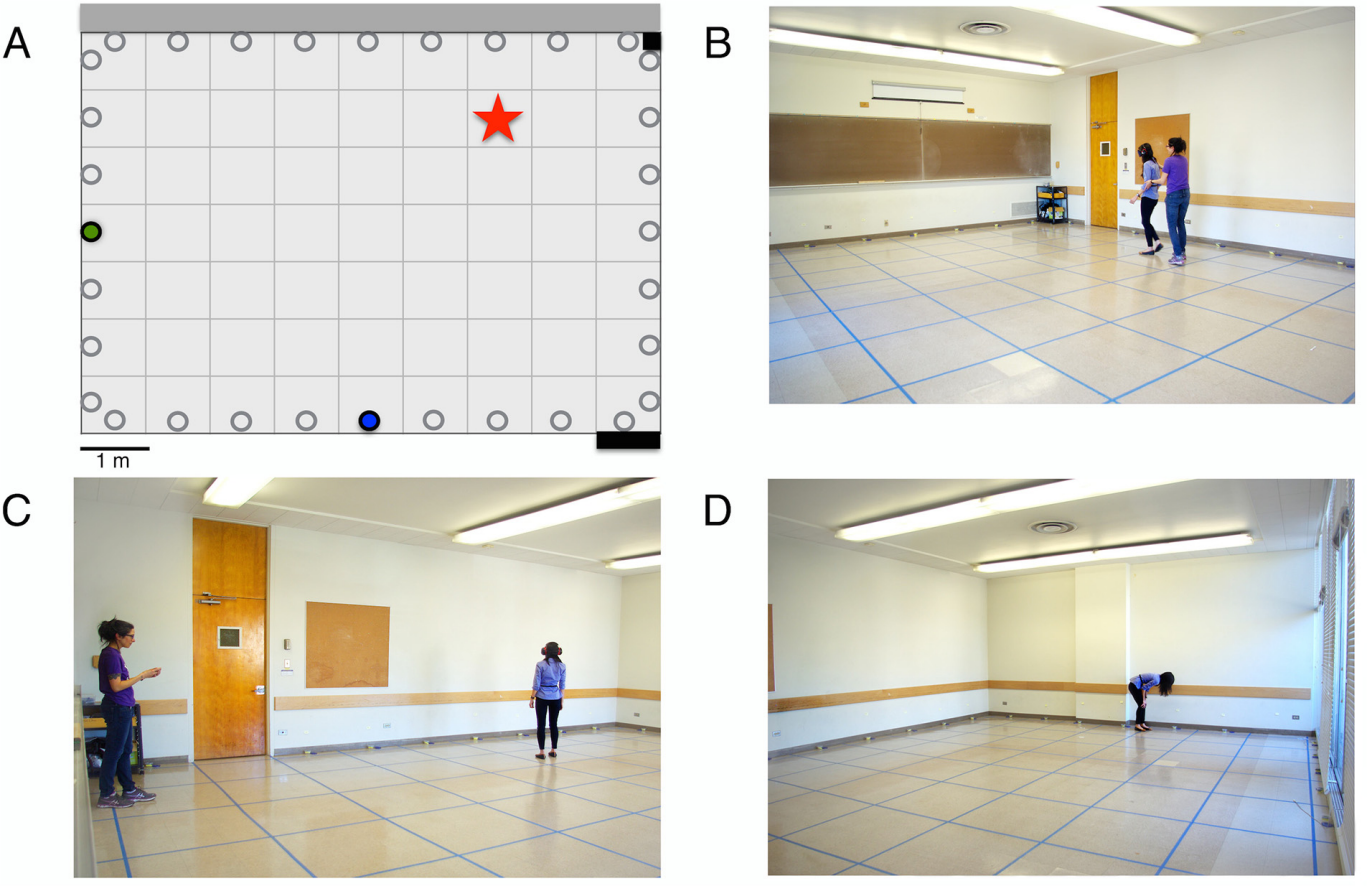
The testing space and the testing procedure. (A) Schematic of test room. Open circles indicate decoy odor receptacles; filled circles indicate receptacles containing odor. Star indicates an example of an arbitrary target location. (B-D) Photographs of the test room in Tolman Hall and the procedure. The test room was divided into 63 quadrants, each outlined in blue adhesive tape. The room contained a small wheeled cart at the entrance, a video camera on a tripod in the opposite corner (not visible in these photos), and a 30.5-cm-high cm high air purifier in a third corner (not pictured). The long wall opposite the entry (visible on the right in D) was glass and overlooked the campus. This wall contained a full-size glass door and two large windows, all of which were opened to air the room between participants but were closed during the test procedure. (B) A researcher disorients the participant by leading her in a meandering path to a previously chosen random target location in the room. During this procedure, the participant is asked three times to point towards the entry location, to ensure that she is disoriented before taking the sensory sample at the target location. (C) The participant takes a one-minute olfactory sample of the air at the target location. (D) Upon completion of the sample phase, the participant removes the sensory covers and is allowed to search for as much time as needed to relocate the target location.

Data collection took place in a large (8.3 m x 6.4 m) room in Tolman Hall (Fig 1). The door entering the room from the hallway was located near the corner of one long wall. The other long wall was a glass wall opening to the outdoors, containing two glass windows and a glass door. All doors and windows were always closed during testing, and no one entered or exited the room during testing. The floor of the room was divided into small square quadrants (0.91 m x 0.91 m), which were marked off using blue adhesive tape. These quadrants were numbered along two axes, with 7 subdivisions on the x axis and 9 on the y axis. We will refer to a location in these coordinates, e.g., the start location was location (1,1) and the farthest quadrant from the start was location (7,9). A line of possible odor-containing receptacles (Glad Mini Round 118 ml containers) was arranged along each wall of the room. These were small, round, lidded plastic storage containers (32 in total), each of which contained a small (2.5 cm x 3.8 cm) sponge (cut pieces of Scotch Brite Heavy Duty Scrub Sponges) that had a rough green scrubbing surface on one side. One sponge and container was placed at the outer edge of each quadrant that was adjacent to a wall. The room contained slate blackboards on one wall, a wheeled cart in the corner by the door, at location (1,1) which was used to hold study materials, a video camera on a tripod in the corner at location (7,1), and a 30.5-cm-tall air purifier in the corner at location (1,9). The room was ventilated but not artificially cooled and was heated only by ambient sunlight. Using readings from the installed thermostat, the mean room temperature during testing was 21.5°C (*SD* = 1.2), with a range from 18.9°to 23.3°C. Because the volatility of odors is affected by temperature and the room temperature varied fairly widely, we examined room temperature as a variable of interest.

The olfactory stimuli used were three specific essential oils: sweet birch oil (*Betula lenta*), anise oil (*Pimpinella anisum*), and clove bud oil (*Eugenia caryophyllata*). These were 100% pure essential oils of these species, obtained from the manufacturer Lhasa Karnak, Berkeley, CA. These essential oils were chosen because they are the standard stimuli used to train dogs in olfactory orientation. Although clove oil is a trigeminal stimulant and, with extensive or repeated exposure, may have an analgesic effect [30], participants' exposure to this stimulus was minimal and, we believe, below the threshold for such analgesia. We found no differences in performance between the 22 participants exposed to this stimulus and the remaining 23 participants.

### Procedure

We measured orientation ability when using different sensory modalities in a within-subjects design, with all participants tested using the same order of conditions. These were: one Test condition, Olfaction (olfactory only, with no visual or auditory access to room stimuli); one Task Validation condition (visual cues only, with no olfactory or auditory access to room stimuli); and one Control condition (with no olfactory, visual or auditory access to room stimuli). Our goal was to compare performance in the Test and Control conditions. In the Test condition, Olfaction, participants were blindfolded and wore earplugs and sound-reducing headphones. In the Task Validation condition, participants wore nose clips, earplugs and sound-reducing headphones. In the Control condition, participants wore all of the sensory covers, blocking olfaction, vision and audition. All participants were tested in the same order of conditions: Test (Olfaction), then Task Validation, then Control, to ensure that the increased familiarity with the room and the procedure would facilitate their performance only in the Control condition and not the Olfaction condition.

All three windows and one door to the room were left open before and after testing, for a minimum of 15 minutes between participants, to clear the odors from the room. We ran a manipulation check, separate from actual testing of participants, to verify that the room was cleared of detectable odors after 15 minutes. This check consisted of a research assistant going into the room and either opening or not opening the odorant containers, placed at locations (1,5) and (4,9). After 15 minutes, the containers were closed if they had been open and the door and windows were opened. After an additional 15 minutes, two naïve research assistants entered the room and were asked whether they could smell any odors and whether they believed that the containers had been opened or not. This procedure was repeated three times, with the containers opened the first two times and closed the third time. No assistant reported smelling any odor on any trial. An additional five naïve volunteers were asked to enter the room on the second trial and report on “whether they could smell anything” and all stated that they could not smell any odors. For each of the first two trials, one assistant thought that the containers had been opened and the other thought that they had not been opened. On the third trial, both assistants thought that the containers had not been opened. Fifteen minutes thus appears to be an adequate time to clear the room of detectable odors.

Upon arrival, participants completed consent materials and all self-report measures while seated in the hallway outside the test room. During this time, the researchers determined the participant’s target locations for each of the three conditions by throwing a 10-sided die. The first two throws determined first the x and then the y coordinate for the Olfaction condition, the second two throws determined x then y for Task Validation, and the third two throws determined x then y for the Control condition. If a throw yielded a number higher than the number of quadrants on that axis, it was disregarded and the die was rethrown. This procedure was completed inside the room; the participant was given no information regarding the target locations.

The 32 sponge-filled containers were already set up in the room when the participant arrived. The two containers at locations (1,5) and (4,9) were taken out of the room, out of view of the participant, and each of these two sponges was impregnated with 30 drops of a single essential oil. Thus, two essential oils were used for each participant, each emanating from one container at one location. This was done while the participant was working on the self-report measures. The oils were always placed on the sponge’s green side, so that the application of the oil did not change its visual appearance; all sponges were placed green side up in the containers. After the essential oils were added, the containers were resealed and brought into the room, to control the timing of odor dispersal. Sponge containers were used immediately after preparation and were used only once, then replaced for each new participant. After being scented and resealed, the two sponge containers were replaced in their locations along the wall, unobserved by the participant, who was still completing self-reports in the hallway. Participants were not informed regarding the number, location, or identity of the odors.

Participants were then brought into the test room. All doors and windows were closed after the participant entered, and remained closed until all three experimental conditions were completed. The air purifier was turned off during testing. Participants were given the instructions (S1 File), and they were then asked to put on a cloth blindfold, insert disposable ear plugs, and put on sound-reducing headphones. Participants were then given one 30-second test experience of being led by the researcher while wearing these sensory covers, to familiarize them with the experience. To lead the participant in all conditions, the researcher walked next to the participant, holding his or her arm below the elbow and touching his or her shoulder, to direct the participant’s movement. After the 30-second familiarization period, participants were placed facing the corner at location (1,1), wearing the sensory covers. One researcher then removed the lids of all the sponge containers one by one, always starting with the container at location (1,1) and moving clockwise around the room. An interval of two minutes was begun when the lid was removed from the second odor-impregnated sponge at location (4,9).

At the end of these two minutes, participants completed the Olfaction condition. In this condition, the goal was to assess whether participants could orient using only olfaction. To eliminate the use of dead reckoning to reorient to the target location, participants were disoriented before being led to the target. To accomplish this, one researcher led the participant at a walking pace circuitously around the room, continuously walking in small circles both to the left and to the right and never walking in a single direction for more than 3 steps in a row, for a total of 2 minutes (Fig 1B). Gentle spinning overwhelms the vestibular signal that would be necessary for dead reckoning, and can be done without resulting in vertigo or dizziness [31]. Pilot testing had indicated that this procedure was adequate to disorient a person wearing blindfold and ear covers, and that walking in this way did not result in vertigo or dizziness; indeed, no participant reported or showed evidence of any such adverse reaction. To verify that participants were indeed disoriented with regard to their location, a second researcher stood at location (1,1) to measure the participant’s state of disorientation. This researcher signaled four successive 30-second intervals with a hand signal. At each of the 30-second intervals, participants were tapped on the shoulder by the first researcher and instructed to point towards the entrance door. The direction in which the participants pointed was recorded on paper by the second researcher. The researchers communicated silently, using only hand signals. After two minutes, the participant was placed at the target location. This was the center of the target quadrant, and participants were always placed facing the wall that contained the entrance door, location (1,1; Fig 1C). Participants stood in their target location for one minute and were instructed to “smell what this location smells like.” After this, participants were led circuitously around the room for one minute to return to the start point, location (1,1).

Participants were then asked to remove all sensory covers and to return to the target location. While participants searched for this location (Fig 1D), their movements were videotaped by one researcher while the second researcher recorded the time taken to choose an estimated target location. Once participants had made this decision, they were asked to continue to stand in that location and to report on whether their choice arose from a conscious awareness of the stimuli at that location or if it was an estimate, based on unconscious awareness. Finally, participants were asked how confident they were about their choice, using a 7-point Likert scale. The quadrant in which the participant was standing was recorded. Participants were then asked to put on visual and auditory covers, and the distance between their choice and the actual target location was measured using a metric tape measure.

Participants were then allowed to remove all sensory covers and return to the starting location (1,1). One researcher then replaced all the lids on all the sponge-containers. Participants were then tested for Task Validation and finally were tested in the Control condition, using a similar procedure but with different sensory covers: while being led to, standing at, and being led from the target location, participants now wore a swimmer’s nose clip in addition to all other covers. In addition, during Task Validation participants were only allowed to remove the blindfold at the target location. Finally, in the Control condition participants stood at the target location without removing any sensory covers.

At the end of the procedure, participants were allowed to see how close their estimate had been to the target for each condition, and any questions were answered. The entire procedure took approximately 50 minutes to complete. Between participants, the air purifier was turned on and both doors on each long wall and both windows were opened, to clear the room of odors. There was always a minimum of 15 minutes of airing between participants. The two containers that had contained odorants were discarded immediately after the participant left, and were replaced with unscented ones for each new participant.

## Results

### Disorientation treatment

To ascertain that participants were disoriented before and after sampling the target location, we analyzed their ability to point to the start location three times (every 30 sec). We categorized points into eight directions (either towards a corner or a wall). In the Olfaction condition, only one participant could still point to the start location by their third attempt, an outcome fully consistent with chance expectation. In the Control condition, 12 participants were able to do this, significantly more than expected by chance (binomial test, *p* = .008), possibly because of increasing familiarity with the space. However, in this condition, those participants who were able to point to the start location did not perform more accurately (as measured by accuracy in estimating distance to target, as described below) when reorienting to the target location, *t*(43) = -1.60, *p* = .12, *M* = 81.2 cm, 95% CI [-21.2, 183.5].

### Reorientation accuracy

**Fig 2:**
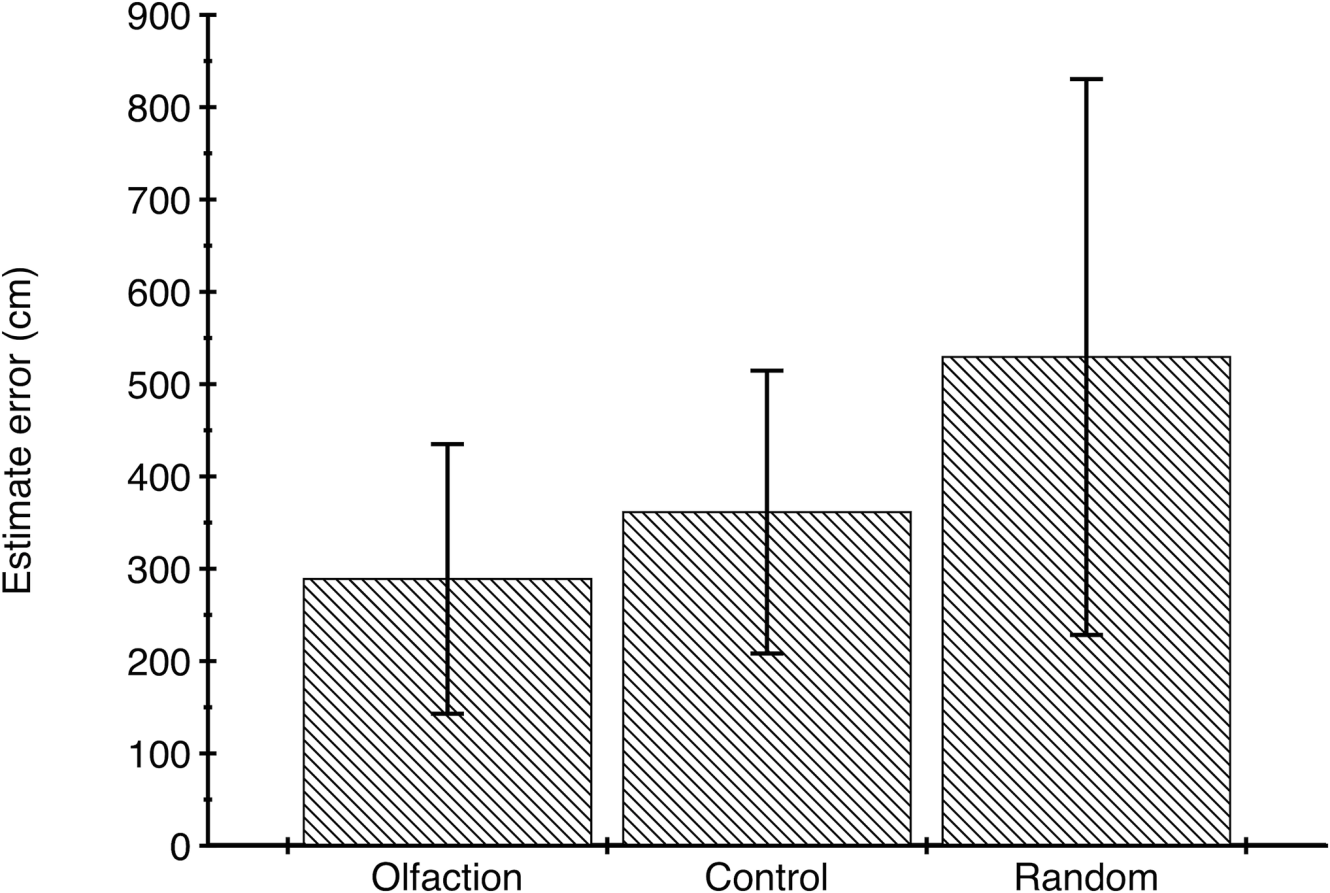
Accuracy of reorientation to the trained location. Mean (±SE) distance (cm) from the target location to the participant’s best estimate of the recalled location for each within-subject condition.

**Fig 3:**
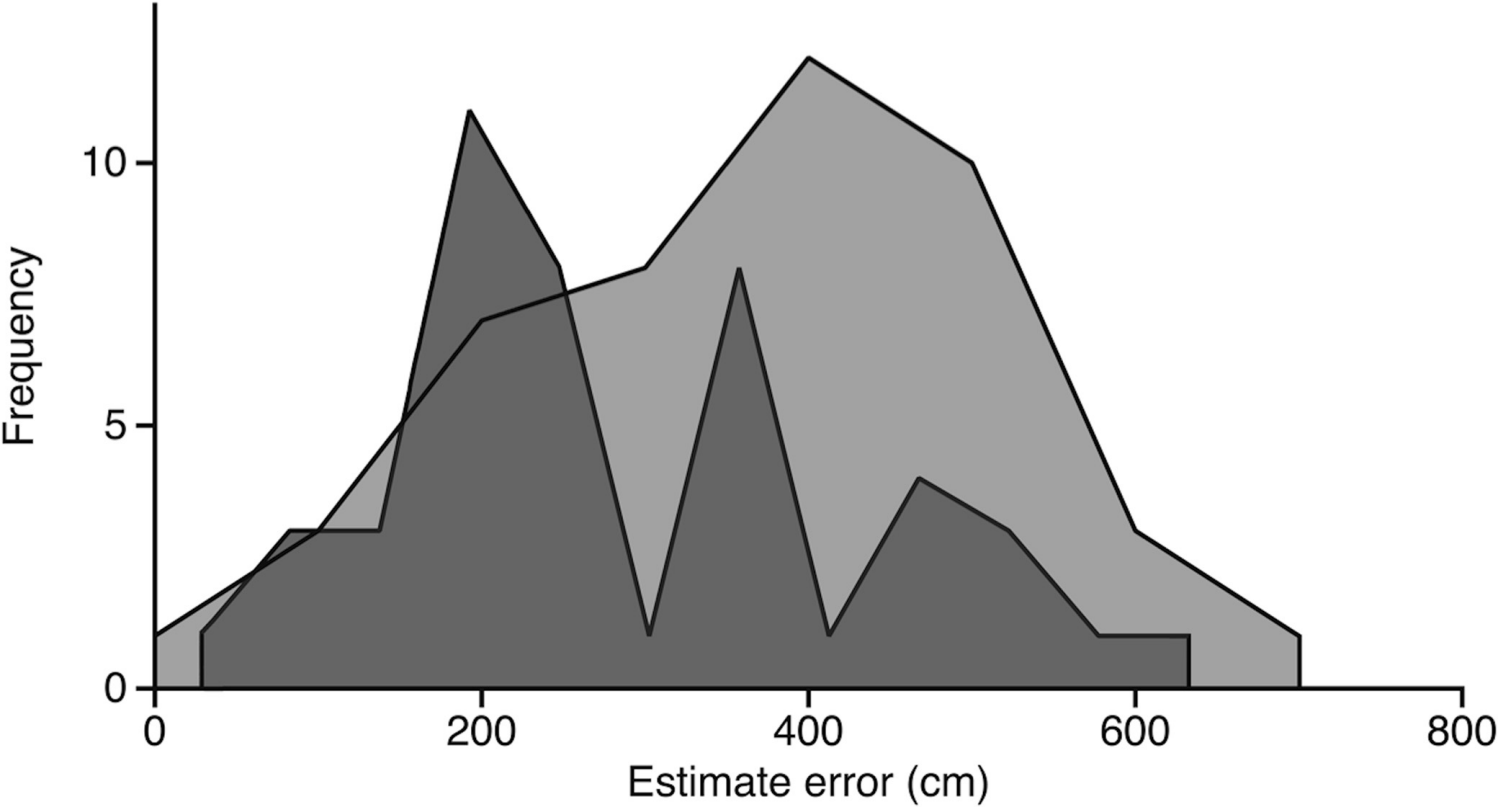
Distribution of estimate error. The frequency distribution of estimate error (in cm) for the Olfaction condition (dark fill) and the Control condition (light fill).

We calculated reorientation accuracy as the distance between the sampled target location and the participant’s subsequent estimate of this location (Fig 2). The mean distance in the Olfaction condition was 289.0 cm (*SD* = 146.0; Fig 3). In the Control condition, it was 361.4 cm (*SD* = 153.2). In the visual Task Validation, as expected, performance was at ceiling (mean distance = 12.3 cm, *SD* = 33.5). We compared target-estimate distance between the Olfaction and Control conditions using repeated-measures ANOVA, in a model that included gender as a between-subjects variable as well as three covariates: participants’ ratings of their confidence in their estimate, latency to locate target in the Olfaction condition and room temperature. Target-estimate distances were significantly larger in the Control condition than in the Olfaction condition, *F*(1, 43) = 4.11, *p* = .049, partial η^2^ = .087, 90% CI [.00, .25]; *M* = 73.9 cm, 95% CI [0.4, 147.4]. No other variables in the model were significant predictors. Thus, reorientation was significantly more accurate when participants had access to odor stimuli compared to the Control condition, in which olfactory information was absent.

Reorientation using olfaction was also significantly more accurate than an average of randomly chosen locations, where the mean target-estimate distance for 1,000 random locations was 529.4 cm (*SD* = 301.1), 95% CI [510.7, 548.1]. This was significantly greater than the mean distance in either condition, and hence the average target-estimate distance in both conditions was significantly smaller than would be observed by chance (Olfaction: *M* = 289.0 cm, 95% CI [245.1, 332.9]; Control: *M* = 361.4 cm, 95% CI = [315.4, 407.4]. Finally, significantly more participants (*n* = 32) estimated more accurately in the Olfaction than the Control condition compared to the number that estimated more accurately in the Control than the Olfaction condition (*n* = 13; binomial test *p* = .003).

Participants spent a mean of 79.9 seconds (*SD* = 62.8) searching for the target location in the Olfaction condition. They spent significantly less time (*M* = 24.9 seconds, *SD* = 18.2) searching in the Control condition, *t*(44) = 6.78, *p* <. 001; mean difference = 54.96, 95% CI [38.6, 71.3]. Search time did not correlate significantly with orientation accuracy, however, as measured by target-estimate distance, in either the Olfaction condition, *r*(45) = .11, 95% CI [-.19, .39], *p* = .49, or the Control condition, *r*(45) = -.15, 95% CI [-.42, .14], *p* = .31.

Participants rated their confidence in their estimate using a 1–7 Likert scale, in which higher scores indicate greater confidence. Average confidence ratings were significantly higher in the Olfaction than the Control condition: Olfaction, *M* = 3.54 (*SD* = 1.41), Control, *M* = 2.26 (*SD* = 1.26); *t*(44) = 5.48, *p* <. 001, mean difference = 1.28, 95% CI [0.81, 1.76). Confidence ratings were not correlated with target-estimate distance within either condition (Olfaction: *r*(45) = .08, 95% CI [-.22, .37], *p* = .60; Control: *r*(45) = -.04, 95% CI [-.26, .33], *p* = .82).

Because the experimental stimuli were volatile chemicals and volatility increases with ambient temperature, we examined the effect of temperature on performance. The average room temperature during testing was 21.5°C (*SD* = 1.2), with a range from 18.9° to 23.3°C. Room temperature did not correlate significantly with performance in the Control condition, *r*(45) = -.049, 95% CI [-.25, .34], *p* = .75, or the Olfaction condition, *r*(45) = -.08, 95% CI [-.22, .36], *p* = .610.

Because of known gender differences in navigational strategy [32], we included gender as a between-subjects variable. The average Olfaction target-estimate distance did not differ significantly between women (*n* = 26; *M* = 282.5 cm, *SD* = 155.5) and men (*n* = 19; *M* = 297.8 cm, *SD* = 135.5); *t*(43) = 0.34, *p* = .73, mean difference = 15.29, 95% CI [-74.5, 105.1].

### Other influences on navigation accuracy

To determine if navigation accuracy was affected by the distance between the odor sources and the target location, we measured the distance between the participant’s target location and each odor source in the Olfaction condition. In the Olfaction condition, there was no correlation between navigation accuracy and distance to the odor at either location (odor at (1,5): *r*(45) = .17, 95% CI [-.13, .44], *p* = .26; odor at (4,9): *r*(45) = .10, 95% CI [-.20, .38], *p* = .51.

The particular combination of odors used for a trial might also affect navigation accuracy in the Olfaction condition. Three combinations were possible: birch and clove (22.2% of trials), anise and clove (26.7%), or anise and birch (51.1%). There were no significant differences in target-estimate distance in the Olfaction condition among these odor combinations, *F*(2, 42) = 0.50, *p* = .61, η^2^ = .02, 90% CI [.00, .10]).

Finally, we examined the relationship between navigation accuracy and participants’ self-ratings of their navigation ability, using the Santa Barbara Sense of Direction (SBSOD) scale. The average score on the SBSOD was 4.2 (*SD* = 1.0) with no significant gender differences. Scores on this scale also did not correlate significantly with performance in either condition: Olfaction: *r*(45) = -.02, 95% CI [-.28, .31], *p* = .92; Control: *r*(45) = .22, 95% CI [-.08, .48], *p* = .15).

## Discussion

Our results indicate that humans can navigate to an arbitrary location that they have learned using only their sense of smell. Participants’ performance was not better when their target location happened to be very close to an odor source, suggesting that participants were not simply orienting toward a single, strong odor but instead were integrating information from both odor sources, attending to the distributions of multiple odors. Participants were more confident in their estimates in the Olfaction condition than in the Control condition, indicating that they had a conscious awareness of their ability to navigate using odors.

The experimental design and the results also ruled out other mechanisms of orientation, specifically, the use of visual or auditory cues in the Olfaction condition and the use of dead reckoning in all conditions. Participants were demonstrably disoriented; that is, they could not point to the entry, before sampling stimuli at the target location. Thus, in the Olfaction condition, they could only have defined the location using olfactory stimuli. Although the percentage of participants who were not disoriented prior to sampling increased in the last condition run (Control), this was not correlated with more accurate estimates. Because the test room had a wall of windows on one side, it is possible that the room temperature was higher on the window side on sunny days. If so, then a participant, with experience, might have unconsciously learned that the entry side was cooler and hence they could remain oriented to this side of the room by pointing towards the cooler side. Even so, this would not facilitate their orientation to an arbitrary target location; and, in fact, accuracy in pointing to the entry was not correlated with navigation accuracy, measured only minutes later.

## Conclusion

The ability to navigate accurately is critical to survival for most species. Perhaps for this reason, it is a general property of navigation that locations are encoded redundantly, using multiple orientation mechanisms, often from multiple sensory systems [33,34]. Encoding the location with independent systems is also necessary to correct and calibrate the accuracy of any one system [35]. As a general principle, then, navigational accuracy and robustness should increase with the number of unique properties exhibited by redundant orientation systems.

Olfaction is perhaps the most universal of these redundant sensory systems, critical for navigation across animal species, even in birds [21]. The use of olfaction in navigation by humans, however, has not been studied in any detail, for at least two reasons. First, it is generally assumed that humans—with so few functional olfactory receptor genes compared, for example, with the laboratory mouse or domestic dog—have a poor sense of smell. Yet olfaction plays a fundamental role in human cognition, where its importance for emotion, memory, and social cognition have been well studied [36,37]. And even though primates, such as humans or squirrel monkeys, have fewer olfactory receptor genes than rodents or dogs, their odor sensitivity can equal or surpass the performance seen in these other species [38]. In addition to sensitivity and despite their relative paucity of olfactory receptor genes, humans can discriminate over one trillion olfactory stimuli, significantly more than the number that can be discriminated by their visual or auditory systems [39]. As Gordon Shepherd suggests, primates, including humans, may accomplish sophisticated olfactory cognitive processing not at the periphery but in central processing, just as the complexity of human language is not limited by the auditory system but by higher order processes using auditory inputs [40].

Despite these advances in human olfactory cognition, it is still assumed that human olfaction functions primarily for discrimination of proximate stimuli, as a “near” sense, and plays little role in spatial orientation to distant stimuli, compared with vision and audition [41]. Yet olfaction offers unique properties for navigation, simultaneously encoding odor mixtures digitally, as unique "odor objects", and as analog representations of odor gradients stretching across a landscape. These dual properties make olfaction an ideal sensory modality for navigation [21]. Perhaps for this reason, the use of chemical stimuli in spatial orientation is nearly universal among animals [42].

This last observation makes it even more surprising that so little is known about the mechanisms humans might use to orient to odors in space, despite anecdotal reports of sailors and early aviators navigating across land and seascapes using odor gradients [12,43]. Instead, there are only reports that humans can track odors [7] and that the visually impaired use odors to recognize locations [44]. Until now there has been no empirical evidence that humans can map an arbitrary location using only odors, an ability we have established in the present study.

This evidence, however, is not a demonstration of true navigation but rather a demonstration that humans can use this sensory modality to map and reorient to a learned location. The recognition of such ability is the first step in the empirical study of human olfactory navigation, just as other orientation mechanisms, such as echolocation in microchiropteran bats or orientation to magnetic fields in sea turtles, were first demonstrated on a small scale under controlled conditions in the lab [45]. Once a novel orientation mechanism has been identified, it can then be studied under natural conditions where its employment may have a significant effect on survival. In the field, the utility of a sensory modality, and hence the weighting of the information derived from it, may be greater than in a laboratory setting [19].Whether the role of olfaction in human navigation would be more important in a small interior space, as in the present study, or whether this advantage would be significantly magnified under more challenging conditions for orientation, such as in the field, awaits future research.

In conclusion, that humans can, with a single one-minute sampling, identify a location by its unique odor mixture and later return to that location with only the olfactory information to inform their subsequent orientation, is a surprising result. It is surprising in part because we assume that humans have a poor sense of smell. It is also surprising because we assume that even if humans had a good sense of smell, they would not be using it for navigation but rather for the discrimination and identification of odors. Yet because so many animals do use olfaction in navigation, perhaps it would be more surprising to find that humans do not. For whatever reason, it is clear that human olfactory navigation has been insufficiently studied. If humans, a species specialized for long-distance walking [46], can employ olfaction in navigation, our results suggest that comparative studies of olfactory navigation across different kinds of vertebrates, whether flying or walking species, could have a rich future.

## Supporting Information

S1 File Participant Instructions. The text that was read to participants before they were tested.
(PDF)
