# Supplementary material for: Olfactory Orientation and Navigation in Humans
Source: PLoS One. 2015 Jun 17;10(6):e0129387. doi: 10.1371/journal.pone.0129387 (PMC4470656; doi:10.1371/journal.pone.0129387)
Supplement: S1 File — The text that was read to participants before they were tested. (PDF) [file pone.0129387.s001.pdf]

# Supporting Information

## Participant Instructions

"In this study we're interested in different ways of navigating. We're going to ask you to find a location in a room using first just your sense of smell, then just vision, then no sense at all. So we'll ask you to put on a blindfold, ear plugs, and a nose clip. These are clean. Then we'll ask you to wait for 2 minutes while we place some odors somewhere in the room. After 2 minutes, I will take your arm and lead you around the room for 2 minutes. Three times during this, I'll tap you on the shoulder; when I do that, try to point toward the door where we came in. I won't tell you whether you're right or wrong in where you're pointing. Then we'll stop at the target location, which was chosen by dice throw and could be any quadrant in the room. You will then have a chance to smell what that location smells like for 1 minute. You can lean forward, crouch down, or turn in circles if you'd like, but please don't take a step in any direction. After 1 minute, I will lead you around the room again and we will end up back in the corner by the door. At that point, I will ask you to remove all the sensory covers and to find your way back to the target location, using your sense of smell. There is no time limit but we will record how long you take, and I will record your movements with the video camera. When you decide on a location, stand there. We'll record your guess and ask you a few questions, then we'll measure how far your guess was from that location. We'll ask you to briefly replace your sensory covers, so that you will not know how close you were to the target location. After this, we will repeat this procedure two more times. The second time, you'll be able to uncover your eyes at the target location; the third time, you won't get to uncover any senses. We'll repeat these instructions during the study; you don't need to remember all of this. Do you have any questions?"
